# Incorporating Advanced New or Recycled Materials in Reinforced Concrete Structures

**DOI:** 10.3390/ma19204265

**Published:** 2026-10-09

**Authors:** Ayman El-Zohairy, Antonio Caggiano

**Affiliations:** 1 Department of Engineering and Technology, East Texas A&M University, Commerce, TX 75429, USA; ayman.elzohairy@etamu.edu; 2 Dipartimento di Ingegneria Civile (DICCA), Chimica e Ambientale, Università degli Studi di Genova, 16145 Genova, Italy; 3 Faculty of Engineering, Universidad de Buenos Aires, Av. Las Heras 2214, Buenos Aires C1127AAR, Argentina

Reinforced concrete continues to be one of the most widely used structural materials worldwide because of its versatility, availability, durability, and ability to accommodate a range of structural forms and loading conditions. However, the construction sector is facing mounting pressure to reduce its consumption of natural resources, minimize waste generation, lower embodied environmental impacts, and improve the long-term performance and resilience of infrastructure. These challenges have stimulated substantial research into advanced and recycled materials that can partially replace conventional constituents, enhance concrete performance, extend structural service life, or introduce new functionalities into reinforced concrete systems [1,2].

Against this background, this Special Issue of *Materials*, titled “Incorporating Advanced New or Recycled Materials in Reinforced Concrete Structures”, was established to provide a focused platform for research addressing the development, characterization, and structural application of innovative materials in reinforced concrete [3]. The scope extends beyond material-level improvements alone and emphasizes the connection between material innovation and structural response. Approaches in which new, modified, or recycled materials influence the strength, stiffness, ductility, cracking behavior, durability, failure mechanisms, and overall performance of reinforced concrete members and structural systems are of particular interest [4].

The motivation for this Special Issue stems from two closely connected needs. The first is the development of higher-performance cementitious and reinforcing systems capable of meeting increasingly demanding structural, durability, and serviceability requirements. Advances in material engineering have introduced numerous opportunities through fiber reinforcement, high-performance cementitious composites, alternative reinforcement systems, material modification techniques, and other emerging technologies. The second need is environmental sustainability, with the incorporation of recycled and waste-derived constituents providing opportunities to conserve virgin resources, divert materials from disposal, and support a more circular approach to construction. However, environmental benefits alone are insufficient for structural implementation; recycled materials must also demonstrate acceptable mechanical behavior, durability, reliability, and compatibility with established structural design principles [5,6].

Accordingly, contributions to this Special Issue were selected based on their relevance to the integration of advanced new or recycled materials into reinforced concrete applications, their technical and scientific contributions, and their potential to advance the understanding of material–structure interactions. Emphasis was placed on studies that move beyond basic material characterization and provide insight into structural behavior through experimental investigation, analytical assessment, numerical simulation, or combinations of these approaches. Studies addressing practical engineering applications, performance enhancement, sustainability, and the development or validation of predictive and design-oriented approaches were also within the intended scope [7,8,9].

The contributions collected in this Special Issue can therefore be viewed within several interconnected thematic areas. One major theme concerns the development and characterization of advanced cementitious and composite materials, with attention to how modifications in material composition translate into improved mechanical or structural performance. A second theme addresses the use of recycled and waste-derived materials in concrete, reflecting the growing importance of resource efficiency and circularity in construction. A third area focuses on structural behavior and performance, including the strength, deformation, cracking, ductility, failure modes, and load-transfer mechanisms in reinforced concrete members incorporating unconventional materials. Complementing these experimental investigations are analytical and numerical modeling approaches used to interpret structural responses, evaluate governing parameters, and support both prediction and design.

Taken collectively, these themes reflect a broader transition in reinforced concrete research from treating sustainability, material innovation, and structural performance as separate objectives to considering them as interconnected aspects of structural–material design. The studies presented in this Special Issue contribute to this transition by examining how innovative material solutions can be translated into measurable structural benefits while addressing the sustainability challenges facing contemporary construction. The following sections summarize the individual contributions and, more importantly, highlight the common findings, emerging research directions, and remaining challenges identified across the collection [10].

The first group of papers addresses “durability” as a central requirement for linking material innovation with durable and low-maintained structures. Almakrab et al. [11] examined several strategies for mitigating the alkali silica reaction (ASR), with an emphasis on supplementary (low-carbon) cementitious materials as well as waste-derived components. The work deals with accelerated mortar-bar tests, demonstrating how metakaolin addition can be particularly effective in reducing expansion by 79–88%, with cement replacement levels between 10% and 30%. The authors also tested waste-glass powders which progressively led to a greater expansion reduction as their dosage increased. The combination of metakaolin and lithium further reduced expansion below ASTM thresholds.

Arowojolu et al. [12] studied transportation concrete exposed to aggressive deicing environments. Experimental tests analyzed the freeze–thaw cycles, electrical resistivity, chloride ingress, and thermogravimetric properties of pavement mixtures. The results showed that the incorporation of supplementary cementitious materials (i.e., ternary systems containing Class C fly ash and silica fume) can substantially improve resistance to severe exposures. Together, both studies [11,12] emphasize that appropriate material selections, along with use of low-carbon components, can help responses under deterioration processes that govern the service life of modern infrastructures.

Within this Special Issue, “resource circularity” is then addressed by studies that investigate the reuse of waste arising both within and outside concrete production. For example, Tsardaka et al. [13] evaluated wastewater (WW) recovered from washing concrete trucks. WW can be used as a replacement for fresh mixing water, and this work showed that its influence depends strongly on the cement chemistry of the mixture. Particularly, it can influence the hydration processes and related kinetics, compressive strength, early-age resistance, and durability performance, as demonstrated via carbonation and chloride-related indicators. The work therefore highlights the possible uses of WW while emphasizing that it cannot be used as an isolated ingredient, but rather as part of the full binder–water system, where its interactions with supplementary constituents may control the mixture performance.

The works of Nov et al. [14,15] considered municipal solid waste (MSW) ash as a potential secondary raw material for concrete. In [14], a controlled laboratory-scale incineration procedure for MSW characterized the resulting ash. They represented approximately 18% of the original MSW mass, containing calcium-rich phases (i.e., anhydrite, alite and calcite). The study investigated their potential to suitably replace cement powders and fine aggregates. In [5], the authors examined the generation of ashes from semi-industrial incinerators. These ashes were again dominated by calcium-rich phases (hatrurite representing the major crystalline fraction) and their use were tested to partial replace the sand in the mixture. These sequences, from laboratory combustion and experimental characterizations [14] to semi-industrial and scaled-up production [15], are particularly relevant to circular-economy implementation approaching industrial practice for the concrete industry.

Other contributions focused on “mechanics” and “numerical tools”. Ma et al. [16] used multiphase inclusion theory and FEM simulations to investigate recycled-concrete models predicting responses in natural stone, ceramic tile, glass, red brick, waste concrete, and aerated brick concrete. The work provides a useful computational mechanics basis for moving beyond empirical (trial-and-error) experimental replacement ratios toward multiphase selections and combinations of recycled aggregates. Liu et al. [17] modeled rubberized concrete under cyclic loading by developing a cohesive-zone interface model that incorporates fatigue-damage accumulation. The model is able to reproduce crack paths, interfacial damage evolution, and fatigue life for beams containing different amounts of rubber replacement rates. These two studies [16,17] demonstrate how mesoscale and multiscale models can explicitly connect constituent properties to larger structural/building scales and are therefore important in complementing experimental tests when new recycled materials need to be investigated.

At the “structural-scale” level, the last work by Válek et al. [18] studied a large mockup test made of prestressed ultra high-performance fiber-reinforced concrete (UHPFRC). Two 10.32 m-span arches assembled from semi-arches were loaded until failure, with first cracking observed at 60 kN and a maximum load of 170 kN. The structural response and subsequent failure were also reproduced using a 3D FEM model with a damage-plasticity formulation. Beyond the specific structural scale configurations, the study is significant in illustrating the progression from advanced material formulation to fabrication, full structural testing, and numerical validation.

Considered together, the eight contributions in this Special Issue underline the common direction of today’s research: to develop concrete incorporating advanced, low-carbon, and recycled materials. It emerges that sustainability cannot be assessed independently from durability, structural integrity/reliability, and mechanical performance [19,20]. These observations also open priorities for future research; for example, the long-term performance under coupled actions, such as mechanical loading, moisture transport, temperature variation, chlorides attack, carbonation, freeze–thaw cycles, and other environmental effects, requires more attention [21,22]. Life-cycle assessment and cost benefits should also be more systematically integrated with mechanical and durability testing. Environmental benefits must be quantified, supporting the introduction, alongside the conventional ultimate limit state (ULS) and serviceability limit state (SLS) of current codes, creating a third design criterion that may be called the “environmental limit state (ELS)” [23,24,25].
